# High-Dose Compound Heat Map for 3D-Cultured Glioblastoma Multiforme Cells in a Micropillar and Microwell Chip Platform

**DOI:** 10.1155/2017/7218707

**Published:** 2017-10-18

**Authors:** Dong Woo Lee, Sang-Yun Lee, Il Doh, Gyu Ha Ryu, Do-Hyun Nam

**Affiliations:** ^1^Department of Biomedical Engineering, Konyang University, Daejeon 35365, Republic of Korea; ^2^Department of Health Sciences and Technology, SAIHST, Sungkyunkwan University, Seoul 06351, Republic of Korea; ^3^Center for Medical Metrology, Korea Research Institute of Standards and Science (KRISS), Daejeon, Republic of Korea; ^4^Office of R&D Strategy & Planning, Samsung Medical Center, Seoul, Republic of Korea; ^5^Department of Neurosurgery, Samsung Medical Center, Sungkyunkwan University, School of Medicine, Seoul 06351, Republic of Korea

## Abstract

Glioblastoma multiforme (GBM) is recognized as the most common and lethal form of central nervous system cancer. To cure GBM patients, many target-specific chemotherapeutic agents have been developing. However, 2D monolayer cell-based toxicity and efficacy tests did not efficiently screen agents due to the pool reflection of in vivo microenvironments (cell-to-cell and cell-to-extracellular matrix interaction). In this study, we used a 3D cell-based, high-throughput screening method reflecting the microenvironments using a micropillar and microwell chip platform to draw a high-dose heat map of the cytotoxicity and efficacy of 70 compounds, with two DMSO controls. Moreover, the high-dose heat map model compared the responses of four 3D-cultured patient-derived GBM cells and astrocytes to high dosages of compounds with respect to efficacy and cytotoxicity, respectively, to discern the most efficacious drug for GBM. Among the 70 compounds tested, cediranib (a potent inhibitor of vascular endothelial growth factor (VEGF) receptor tyrosine kinases) exhibited the lowest cytotoxicity to astrocytes and high efficacy to GBM cells in a high-dose heat map model.

## 1. Introduction

**Figure 1:**
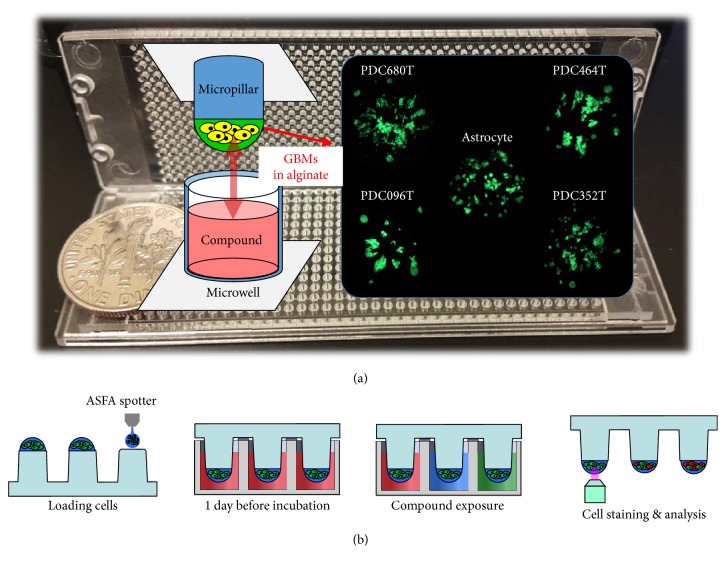
3D cell-based high-throughput screening chips. (a) Photo and schematic view of micropillar and microwell chip platform. Green dots are 3D-cultured astrocytes and glioblastoma multiforme (GBM) cells in alginate spot on the micropillar. (b) Schematic view of the experimental procedure. Cells are dispensed and immobilized in alginate onto the top of the micropillars and dipped in the microwells containing growth media for 1-day culture by sandwiching the micropillar and microwell chips. Compounds are dispensed into the microwells and cells are exposed to the compounds by moving the micropillar chip to a new microwell chip. 3D-cultured cells are stained with Calcein AM, and the dried alginate spot on the micropillar chip is scanned for data analysis.

Glioblastoma multiforme (GBM) is the most common, aggressive, and lethal primary malignant brain tumor that stems from astrocytes. These tumors are usually highly malignant because the cells can metastasize from the primary tumor without detection and invade the surrounding normal brain tissue to form new tumor “satellites” that lead to tumor recurrence [1]. The current standard of care is surgical resection coupled with ionizing radiation (IR) and the chemotherapeutic agent temozolomide (Temodar®, Temodal®, TMZ) [2, 3]. However, this treatment only provides patients with GBM a 12–14-month survival period after diagnosis [2, 3]. Despite aggressive surgical resection and chemotherapy, almost all patients with GBM present with tumor recurrence. Thus, many target-specific or general-chemotherapeutic agents have been developed to cure patients with GBM. Although some of the compounds exhibit good efficacy toward GBM, the resulting cytotoxicity of normal glial cells in the central nervous system has been an issue. To measure cytotoxicity of compounds in normal glial cells, neural stem cells or astrocytes are used [4, 5]. Astrocytes are the most abundant member of the glial family and have a wide range of adaptive functions in the central nervous system (CNS). They interact with neurons, provide structural, metabolic, and trophic support, participate in synaptic activity, mediate ionic and transmitter homeostasis, and regulate blood flow [6, 7]. Since astrocytes play an important role in the CNS, treatment-induced toxicity of the CNS remains a major cause of morbidity in patients with cancer [8]. Thus, a high-dose heat map model comparing the responses of high-dose compounds on astrocytes and GBM cells is required to validate the most efficacious drugs toward GBM. Previous high-dose heat map models using 2D cell-based high-throughput screening are well developed [9, 10]. However, because 2D cell-based assay does not fully reflect in vivo microenvironments (cell-to-cell and cell-to-extracellular matrix interaction), a 3D cell-based assay was used to screen compounds [11–15], including our previously developed system [13–17]. Especially, 3D cultured astrocyte and GBMs show more in vivo like model [18–20]. Thus, assay based on 3D cultured astrocyte and GBMs with high-throughput manner may give new potential to screen GBM target agents. Our previous system [13–17] shows successfully data of 3D cell-based assays with high-throughput manner by comparing their own data with 2D cell-based assay [13], gene [14], and clinical data [17]. By applying the abovementioned quantitative 3D-cultured cell-assay platform, astrocytes and patient-derived GBM cells were 3D-cultured and screened to select the most represented compounds that were not cytotoxic to normal brain cells and were particularly efficient for patient-derived GBM cells.  Figure 1 shows 3D cell-based high-throughput screening chips culturing three-dimensionally four GBM cells and astrocyte. Since TMZ is a representative drug used in the treatment of patients with GBM, it was used as a control compound to verify the high-dose heat map. By comparing TMZ with 69 other compounds, compounds in the high-dose heat map were tested for cytotoxicity and efficacy in GBM cells.

## 2. Materials and Methods

### 2.1. Astrocyte and Patient-Derived Cell Culture

We purchased NHA-astrocyte AGM (LONZA, Cat. number cc-2565). Astrocyte was cultured with ABM Basal media (LONZA, Cat. number cc-3187) added with AGM SingleQuot Kit Suppl.&Growth Factors (LONZA, Cat. number cc-4123). Patient-derived GBM cells were obtained from GBM patients who underwent brain tumor removal surgery at the Samsung Medical Center (Seoul, Korea). Informed consent was obtained from all patients. Following a previously reported procedure [13], surgical samples were enzymatically dissociated into single cells. Four patient-derived cells were obtained from four GBM patients. Dissociated GBM cells were cultured in cell culture flasks (from Eppendorf, T-75) filled with Neurobasal A (NBA) conditioned media. The NBA conditioned media comprised N2 and B27 supplements (0.53 each; Invitrogen) and human recombinant bFGF and EGF (25 ng/ml each; R&D Systems), hereafter, referred to as NBE condition media. Cell flasks were placed in a humidified 5% CO_2_ incubator (Sheldon Mfg., Inc.) at 37°C. The cells were routinely passed every 4 days at 70% confluence. For the experiment, the cell suspensions were collected in a 50 ml falcon tube from the culture flask. GBM cells were then suspended in 5 mL of NBE condition media. After centrifugation at 2000 rpm for 3 min, the supernatant was removed, and the cells were resuspended with NBA conditioned media to a final concentration of 10 × 10^6^ cells/mL. The number of cells in the NBA conditioned media was calculated with the AccuChip automatic cell counting kit (Digital Bio, Inc.). The rest of the cells were seeded at a concentration of 2 × 10^6^ cells in a T-75 flask containing 15 mL of NBA conditioned media.

### 2.2. Chip Layout and Experimental Procedure

**Figure 2:**
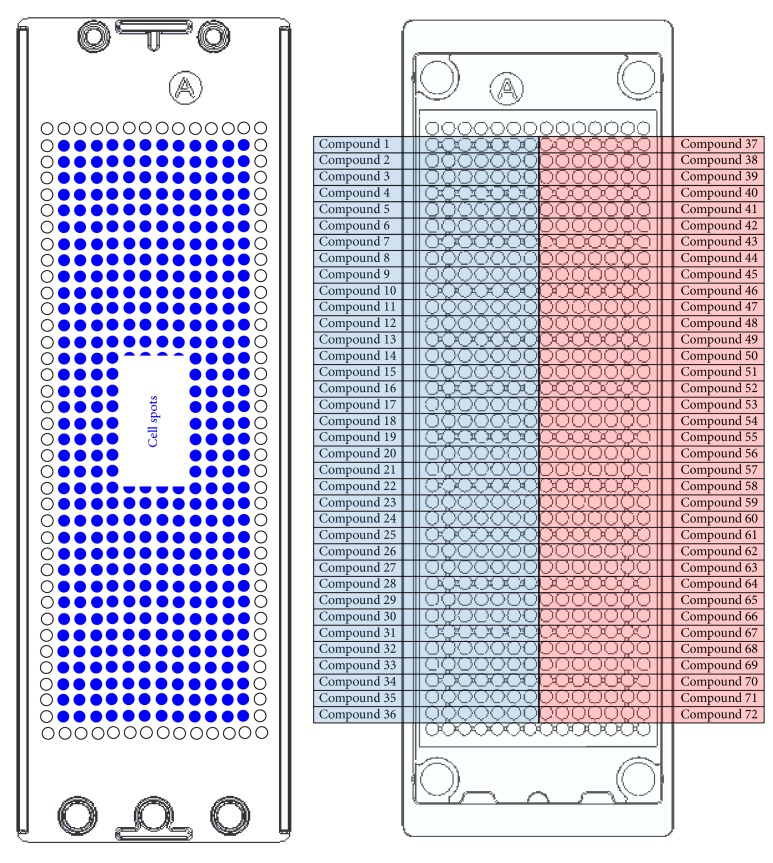
Chip layout for high-dose 72 (including 2 DMSO controls)-compound heat map. Each compound has seven replicates.

The basic layout of the micropillar and microwell chip for a 12-compound screening is shown in Figure 2. The microwell chip is divided into 72 lines, and each line has 6 microwells for replicates. For compound analysis, approximately 100 cells (patient-derived GBM cells) in 50 nL with a 0.75% alginate concentration by volume (0.75 w/w) were automatically dispensed onto a micropillar chip by using ASFA™ Spotter ST (Medical & Bio Device, South Korea). The ASFA Spotter ST uses a solenoid valve (The Lee Company, USA) for dispensing the 50 nL droplets of the cell-alginate mixture and 1 *μ*L of media or compounds. After dispensing the cells, as shown in Figure 1(b), the micropillar chip containing human cells in alginate was sandwiched (or “stamped”) with the microwell chip for 3D cell culture and compound efficacy tests. A single chip can screen 72 compounds with 6 replicates simultaneously. A micropillar chip with alginate dispensed on each pillar spot and the microwell chip containing 72 compounds are shown in Figure 2. The micropillar chip with cells dispensed is stamped together with its complementary microwell chip comprising 532 wells that are 1.2 mm in diameter. One microliter of growth media was dispensed into each microwell. The micropillar and microwell chip in the combined form are shown in Figure 1(b). After 1 day of incubation at 37°C to stabilize the cells, the micropillar chip containing the cells was moved to a new microwell chip filled with various test compounds. Next, the combined chips were incubated for 3 days, as shown in Figure 1(b). Cell viability against the compounds was measured with Calcein AM live cell staining dye (4 mM stock from Invitrogen), which stains viable cells with green fluorescence. The staining dye solution was prepared by adding 1.0 *μ*L of Calcein AM (4 mM stock from Invitrogen) to 8 mL staining buffer (MBD-STA50, Medical & Bio Device, South Korea). To measure cell viability quantitatively after staining the alginate spots, cells on the micropillar chip were scanned. As shown in Figure 1(a), scanned images were obtained with an automatic optical fluorescence scanner (ASFA Scanner ST, Medical & Bio Device, South Korea).

### 2.3. Workflow of High-Dose Compound Heat Map

**Figure 3:**
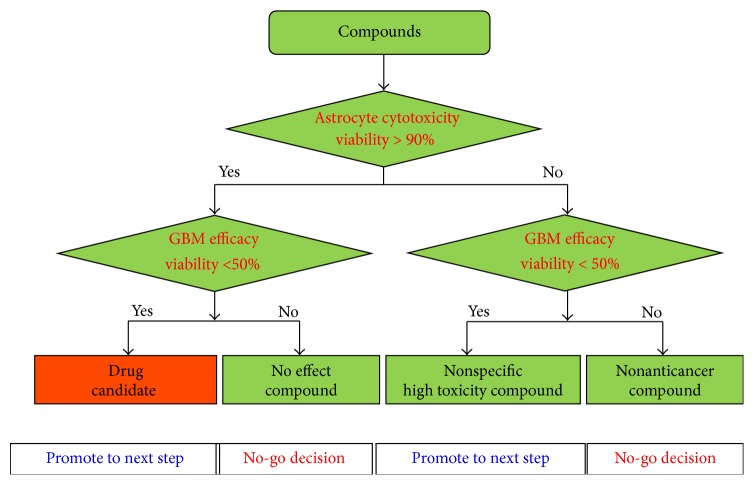
High-dose compound heat map for selecting low cytotoxicity and high efficacy compound.

The high-dose compound heat map model for measuring cytotoxicity and efficacy in GBM cells was quantified using a 3D cell-based screening using a micropillar and microwell chip. The workflow of high-dose compound heat map is shown in Figure 3. 72 compounds with high dosage, including two DMSO controls, were screened against astrocytes and GBM cells for measuring toxicity and efficacy, respectively. Based on astrocyte cytotoxicity and efficacy of GBM cells, compounds were divided into four groups. For this heat map, we selected the most promising compounds that exhibited less cytotoxicity toward astrocytes and high efficacy toward GBM cells.

## 3. Results and Discussion

To determine the cytotoxicity against normal glial cells, we treated astrocytes (represented as normal glial cells) to high dosages (20 *μ*M) of 72 compounds. Among the 72 compounds tested, TMZ is one of most popular drugs for GBM. The lipophilic nature of TMZ permits it to penetrate the blood-brain barrier and, thereby, allows it to be administered orally. It has also been approved by the US FDA for use in the treatment of refractory anaplastic astrocytoma in adults since 1999 and in newly diagnosed adult patients with GBM since 2005. Thus, we used TMZ as a control compound for comparing cytotoxicity of compounds toward astrocytes. We evaluated cytotoxicity of other compounds in comparison to TMZ and evaluated the efficacy of these compounds with patient-derived GBM cells.

### 3.1. Toxicity of High-Dose Compounds in Astrocytes

**Figure 4:**
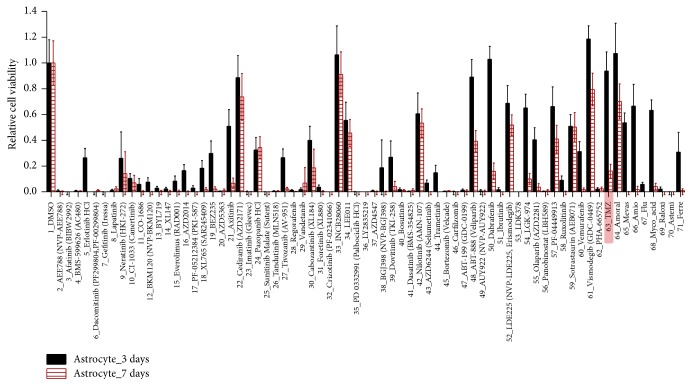
Relative cell viability of astrocytes after exposure to 70 compounds after the 3- and 7-day compound treatment. Cell viabilities are calculated from 3D cell size.

The viability of astrocytes after exposure to 72 compounds (including 2 DMSO control) after the 3- and 7-day treatment is shown in Figure 4. 16 compounds, including TMZ, exhibited high astrocyte viability and increased to >50% after the 3-day compound treatment. On the seventh day, only 7 compounds exhibited >50% astrocyte viability: cediranib, INCB28060, nilotinib, LDE225, sotrastaurin, vismodegib, and amoral exhibited low toxicity (with astrocyte viability >50%). However, TMZ, which is widely known for its low cytotoxicity, exhibited high toxicity (with astrocyte viability < 18%) after the 7-day compound treatment. 20 uM of TMZ is high dose and TMZ shows high toxicity for long-day culture (7 days). Thus, based on the 3-day compound treatment, we evaluated the cytotoxicity of the compounds by comparing the viability of astrocyte after exposure with other compounds and with TMZ (over 90%). Among 16 compounds, 7 exhibited similar astrocyte viability to TMZ, cediranib, INCB28060, ABT-888, dabrafenib, vismodegib, and amoral and therefore may be good candidates to test the efficacy against patient-derived GBM cells.

### 3.2. Efficacy Test of 70 Compounds for Patient-Derived GBM Cells

**Figure 5:**
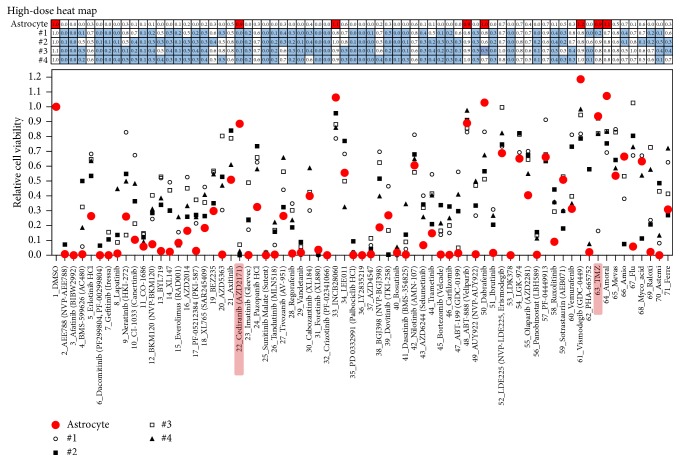
High-dose compound heat map. Astrocytes and four patient-derived glioblastoma multiforme (GBM) cells were treated with 70 compounds for 3 days. In the high-dose heat map (table over graph), red solid filling denotes astrocytes with viability >90% and blue solid filling denotes GBM cells with viability <50%.

The viabilities of astrocyte and four patient-derived GBM cells after the 3-day compound treatment are shown in Figure 5. Most of the targeted compounds exhibited high efficacy with 20 uM dosages. By comparing efficacy of GBM cells and cytotoxicity of astrocytes, most of the compounds exhibited nonspecific, high toxicity for both GBM cells and astrocytes. Among seven nontoxic compounds, INCB28060, ABT-888, vismodegib, and amoral did not suppress the patient-derived GBM cells. Thus, they may be ineffective compounds in treating GBM. TMZ, cediranib, and dabrafenib exhibited low GBM cell viability in more than one patient-derived GBM cell. In particular, cediranib exhibited high efficacy in all four patient-derived GBM cells and exhibited no cytotoxicity toward astrocytes, while TMZ showed high efficacy in only #1 GBM cell among the four patient-derived GBM cells. Previous studies [21, 22] have shown that cediranib is a potent oral inhibitor of vascular endothelial growth factor (VEGF) receptors and demonstrates improved progression-free survival in an uncontrolled phase II study of patients with recurrent glioblastoma. The drug is administered orally and once daily and has a manageable side-effect profile. In addition, it has potent antiedema and steroid-sparing effects that may improve the quality of life of patients with GBM. Several clinical trials are ongoing testing cediranib in patients with gliomas. Through this 3D cell-based high-dose heat map model, we could also identify cediranib as a valid compound for proof-of-concept of heat map model. So, this 3D cell-based high-dose heat map could narrow down drug candidates for GMBs. As further study, we need to draw dose response curve to measure IC_50_ in GBMs about agents showing low toxicity and high efficacy.

## 4. Conclusion

We used a 3D cell-based, high-throughput screening technique using a micropillar and microwell chip platform to determine the effects of high dosage compounds on the cytotoxicity and efficacy on astrocytes and GBM cells, which were graphically represented in a high-dose heat map model. Although some compounds exhibited good efficacy toward GBM, cytotoxicity toward normal glial cells in the central nervous system was an impending issue. Thus, we used a high-dose heat map model and considered both cytotoxicity and efficacy by comparing the response of high-dose compound on 3D-cultured GBM cells and astrocytes to screen the most efficacious drugs for GBM. Seventy compounds with dosage of 20 uM, including specific-target general-chemotherapeutic agents, were used against four patient-derived GBM cells and astrocytes to compare the cytotoxicity and efficacy of the compounds. For simulating an in vivo microenvironment, GBM cells and astrocytes were encapsulated with alginate and 3D-cultured in a micropillar and microwell chip platform. Among the 70 compounds tested, cediranib exhibited the lowest cytotoxicity toward astrocyte and showed high efficacy toward GBM. Thus, in early stage of the drug development, the micropillar and microwell chip platform could culture patient-derived GBMs with 3D manner and be used for screening toxicity and efficacy of many lead compounds targeted GBMs before animal tests or clinical trials.
